# Annual Body Mass Index Gain and Risk of Gestational Diabetes Mellitus in a Subsequent Pregnancy

**DOI:** 10.3389/fendo.2022.815390

**Published:** 2022-03-25

**Authors:** Sho Tano, Tomomi Kotani, Takafumi Ushida, Masato Yoshihara, Kenji Imai, Tomoko Nakano-Kobayashi, Yoshinori Moriyama, Yukako Iitani, Fumie Kinoshita, Shigeru Yoshida, Mamoru Yamashita, Yasuyuki Kishigami, Hidenori Oguchi, Hiroaki Kajiyama

**Affiliations:** ^1^ Department of Obstetrics and Gynecology, Nagoya University Graduate School of Medicine, Nagoya, Japan; ^2^ Department of Obstetrics, Perinatal Medical Center, TOYOTA Memorial Hospital, Toyota, Japan; ^3^ Division of Perinatology, Center for Maternal-Neonatal Care, Nagoya University Hospital, Nagoya, Japan; ^4^ Department of Obstetrics and Gynecology, Fujita Health University School of Medicine, Toyoake, Japan; ^5^ Data Science Division, Data Coordinating Center, Department of Advanced Medicine, Nagoya University Hospital, Nagoya, Japan; ^6^ Kishokai Medical Corporation, Nagoya, Japan

**Keywords:** GDM, BMI gain, interpregnancy care, previous history, recurrence

## Abstract

**Introduction:**

Weight change during the interpregnancy is related to gestational diabetes mellitus (GDM) in the subsequent pregnancy. In interpregnancy care/counseling, the timeframe for goal setting is important, while the timing of the next conception is unpredictable and preventing age-related body weight gain is difficult. This study aimed to investigate the association between annual weight gain during the interpregnancy, which provide clearer timeframe, and GDM in subsequent pregnancies.

**Methods:**

This multicenter retrospective study was conducted by collecting data on two pregnancies of the same women in 2009–2019. The association between annual BMI gain and GDM during the subsequent pregnancy was examined.

**Results:**

This study included 1,640 pregnant women. A history of GDM [adjusted odds ratio (aOR), 26.22; 95% confidence interval (CI), 14.93–46.07] and annual BMI gain (aOR, 1.48; 95% CI, 1.22–1.81) were related to GDM during the subsequent pregnancy. In the women with a pre-pregnant BMI of <25.0 kg/m^2^ and without GDM during the index pregnancy, an annual BMI gain of ≥0.6 kg/m^2^/year during the interpregnancy were associated with GDM in subsequent pregnancies; however, in the other subgroups, it was not associated with GDM in subsequent pregnancies.

**Conclusions:**

For women with a pre-pregnant BMI of <25.0 kg/m^2^ and without GDM during the index pregnancy, maintaining an annual BMI gain of <0.6 kg/m^2^/year may prevent GDM during the subsequent pregnancy.

## Introduction

Gestational diabetes mellitus (GDM) is defined as a diabetes diagnosed in the 2^nd^ or 3^rd^ trimester of pregnancy that was not clearly overt diabetes prior to gestation (1). The incidence is reported to be 12-18% of all pregnancies (2), and the recurrence rate of GDM is as high as 30–70% in a subsequent pregnancy (3–5). Women with a history of GDM have an increased risk of type 2 diabetes mellitus (T2DM) (6–9), metabolic syndrome (10, 11), and cardiovascular disease later in life (12–14). Women with recurrent GDM are reported to have a higher risk of developing T2DM than those with a single event (15). In addition to adverse maternal effects, children of women with GDM are at an increased risk of abnormal glucose metabolism and adiposity (16–18), as well as attention-deficit/hyperactivity disorder (19, 20). Thus, there is an urgent need to establish strategies to prevent GDM; however, there are currently no concrete recommendations for the prevention of GDM.

Interpregnancy care/counseling is well known for its beneficial role in the women’s health and subsequent pregnancy outcomes (21–23). In addition to a history of GDM, being overweight/obese (body mass index [BMI] ≥25.0 kg/m^2^) is a risk factor for developing GDM in a subsequent pregnancy (3, 24–27). Evidence suggests that BMI changes between the index and subsequent pregnancy is also a risk factor for GDM during the subsequent pregnancy (3, 28). Previous meta-analyses and systematic reviews have suggested that interpregnancy BMI gain is associated with higher risk of GDM during the subsequent pregnancies (29–31). The overall interpregnancy BMI gain is certainly a valuable indicator for detecting high-risk for GDM at the first visit for subsequent pregnancy; however, a total interpregnancy BMI change is not a suitable indicator for the prevention of GDM in a subsequent pregnancy. First reason why the total BMI gain is not suitable for prevention is the difficulty in preventing age-related weight gain, as reported previously (32). Recent longitudinal studies have reported that the mean age-related annual weight gain in women younger than 50 years is approximately 0.5 kg/year (33–35). For Japanese women of average height (157.9 cm), the implied age-related annual BMI gain is 0.2 kg/m^2^/year. Second reason is most women do not plan and expect when they will have another baby just after childbirth in the index pregnancy. Considering the difficulties in compensating for this age-related weight gain and unpredictability of the next conception, goal-setting based on total BMI changes during the interpregnancy period can be ambiguous.

One of the most commonly recommended frameworks for goal-setting is the SMART goal model, which is an acronym for Specific, Measurable, Attainable, Relevant, and Time-related (36). While formulating SMART goals, it is important to assess attainability and the timeframes. The concept of “annual BMI change” can provide a more realistic goal-setting process and clearer timeframes. It has already been reported in many medical fields, including oncology (37, 38), diabetes mellitus (39, 40), obstructive sleep apnea (41), and cardiovascular disease (42). Recently, we have also reported that it would be helpful in the interpregnancy care/counseling for hypertensive disorders of pregnancy (HDP) (43); however, no reports have focused on the association between annual BMI changes and GDM.

Thus, this study aimed to evaluate whether an annual BMI gain of ≥0.2 kg/m^2^/year (natural gain) during the interpregnancy period was associated with the risk of GDM during the subsequent pregnancy.

## Materials and Methods

### Study Population

**Figure 1:**
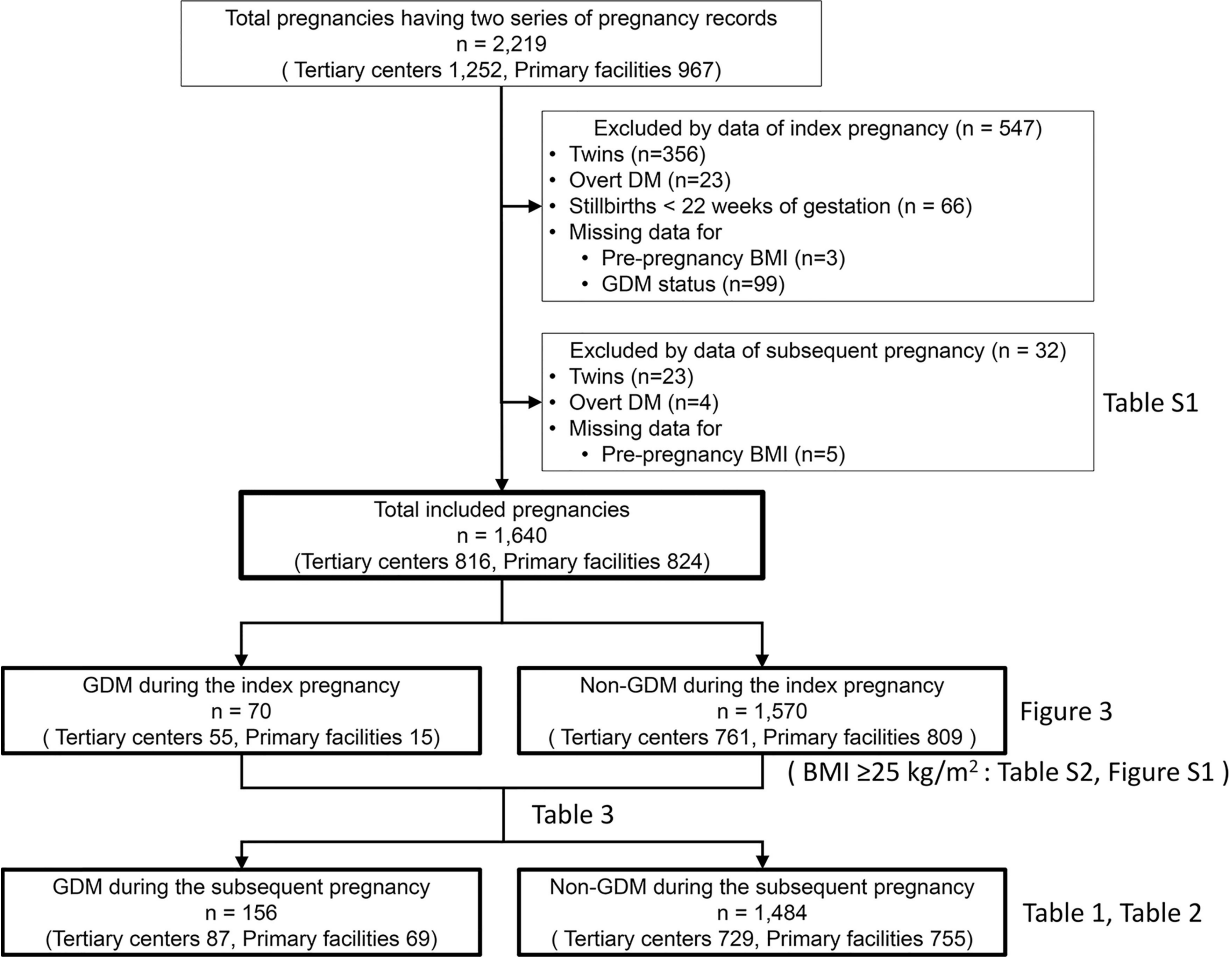
Flow chart of the study participants. Clinical data of 2,219 patients who delivered at two tertiary care centers and 12 primary maternity care units and had available medical records on the index and subsequent pregnancies. A total of 1,640 patients were eligible for this study after excluding 547 and 32 women based on the index and subsequent pregnancy status, respectively. DM, diabetes mellitus; BMI, body mass index; GDM, gestational diabetes mellitus.

This multicenter retrospective study used electronic medical record data of pregnant women aged ≥15 years who delivered at two tertiary centers in Aichi Prefecture (Nagoya University Hospital and TOYOTA Memorial Hospital) or 12 private maternity facilities (Kishokai Medical Corporation located in Aichi and Gifu Prefectures) from 2009 to 2019. Women who had medical records available for both the index and subsequent pregnancies were included. We assessed the medical records directly and ascertained the data, including laboratory tests, if necessary. The exclusion criteria were as follows: pre-pregnancy diabetes mellitus (overt DM), multiple pregnancies, stillbirth before 22 weeks of gestation, and missing data on maternal pre-pregnancy BMI and GDM status (**Figure 1**). Women who developed GDM in a subsequent pregnancy were allocated into the GDM group, while those who did not were allocated into the non-GDM group.

### Definitions of the Variables

Women with pre-pregnancy DM, a hemoglobin A1_C_ (HbA1c) level of ≥6.5% (48 mmol/mol), or a fasting plasma glucose level of ≥126 mg/dL during pregnancy were defined as having overt DM. Based on the clinical recommendation by the Japan Society of Obstetrics and Gynecology (44, 45), GDM was diagnosed based on a two-step approach. First, the casual blood glucose test or a non-fasting 50-g blood glucose challenge test was performed between 24 and 28 weeks of gestation with a cutoff value of 100 mg/dL or a cutoff value of 140 mg/dL, respectively. Second, a 75g oral glucose tolerance test (OGTT) was performed for the women with a positive screening test. Third, GDM was diagnosed when any of the following plasma glucose values were met (1): the 75g OGTT result was a fasting plasma glucose level of ≥92 mg/dL or the 1-h and 2-h plasma glucose levels were ≥180 mg/dL or ≥153 mg/dL, respectively. Assisted reproductive technology (ART) was defined as conception after *in vitro* fertilization or intracytoplasmic sperm injection. Gestational age (GA) was routinely estimated by expected date of delivery (EDD) determined based on the last menstruation cycle and the measurement of the crown–rump length by ultrasonography. In ART pregnancies, EDD was determined using the age of the embryo and the date of transfer. Light-for-date and heavy-for-date were diagnosed using the Japanese standards for birth weight according to the pregnancy durations (≥90^th^ percentile and <10^th^ percentile, respectively) (46, 47). Macrosomia is defined as newborns whose weighs exceed 4,000 g regardless of his or her gestational age (47).

**Figure 2:**
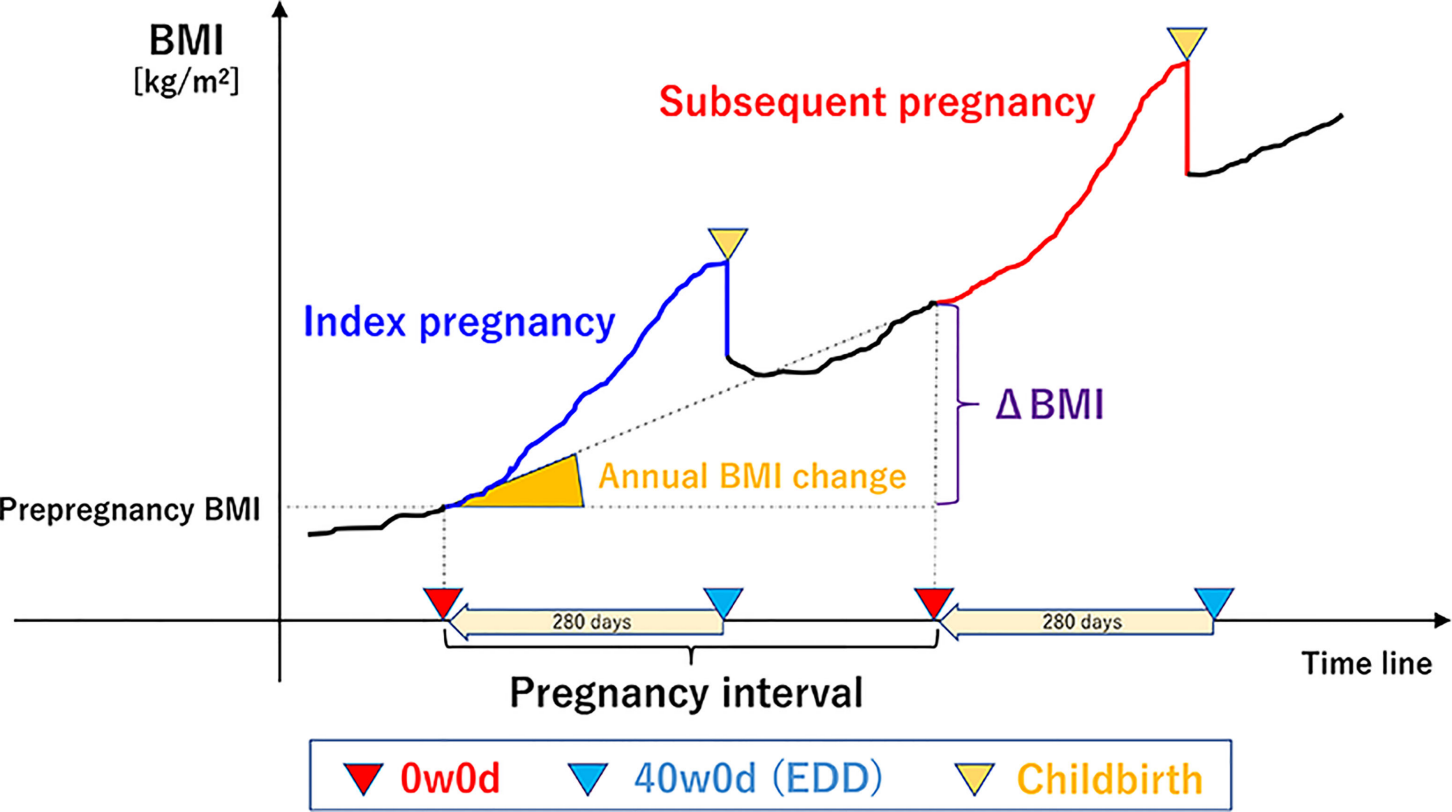
Overview of the definitions of terms. (the reference 43, Tano S et al. Sci Rep,11(1), 22519,2021, Springer Nature). We defined inter-pregnancy BMI change (ΔBMI) as a pre-pregnancy BMI change between the index pregnancy and the subsequent pregnancy. The pregnancy interval was defined as the interval from the EDD of the index pregnancy to that of the subsequent pregnancy, which is equal to the interval between the two gestations. The annual BMI change was calculated as follows: ΔBMI/pregnancy interval. BMI, body mass index; 0w0d, 0^0/7^ weeks of gestation; 40w0d, 40^0/7^ weeks of gestation; EDD, expected date of delivery.

We used the self-reported maternal pre-pregnancy body weight and height obtained during routine practice to calculate the BMI (kg/m^2^) (weight in kg divided by square of the height in m^2^). The calculated BMIs were categorized as <25.0 or ≥25.0 kg/m^2^ according to the World Health Organization’s classifications and previous study (28, 48). As shown in **Figure 2**, we defined interpregnancy BMI change (ΔBMI) as a change in pre-pregnancy BMI from the index pregnancy to the subsequent pregnancy, as previously reported (28). The pregnancy interval was defined as the interval between the two gestations, which is equal to the interval from EDD of the index pregnancy (EDD^index^) to that of the subsequent pregnancy (EDD^subsequent^): (EDD^subsequent^ – 280 days) – (EDD^index^ – 280 days). The annual BMI change was calculated as follows: ΔBMI/pregnancy interval. The annual BMI change during the interpregnancy period was categorized into 5 groups: <0.0 kg/m^2^/year [weight loss], ≥0.0–<0.2 kg/m^2^/year [natural gain, reference], ≥0.2–<0.6 kg/m^2^/year, ≥0.6–<1.0 kg/m^2^/year, and ≥1.0 kg/m^2^/year (43). A gain of 0.2 kg/m^2^/year has been considered a natural annual BMI change (34, 35); gains of 0.6 and 1.0 kg/m^2^/year are equivalent to increments of approximately 1.5 and 2.5 kg/year in the weights of women of average height (157.9 cm), respectively. Gestational weight gain was defined as the change between pre-pregnancy body weight and that before delivery.

### Statistical Analysis

The clinical characteristics and parameters (**Table 1**) of the GDM and non-GDM groups were compared using the Fisher’s exact test, χ^2^ test, Student’s *t*-test, Welch’s *t*-test, or Mann–Whitney U test as appropriate. Crude and adjusted odds ratios (aORs) for GDM during the subsequent pregnancy were calculated using univariable and multivariable logistic regression analyses. Variables used in the univariable and multivariable analyses were selected based on previous studies (26–28, 49–52): maternal age of ≥35 years, pre-pregnancy BMI of ≥25.0 kg/m^2^, the presence of GDM, macrosomia during the index pregnancy, and a parity of ≥2 in the subsequent pregnancy. In addition, insulin use during the index pregnancy was added as a variable for the subgroup analysis of GDM recurrence (26). The annual BMI gains were classified into five categories based on their distributions, as mentioned above, and a multivariable analysis was performed to determine how the aOR changed with specific annual BMI changes.

**Table 1:** Baseline characteristics and perinatal outcomes.

	GDM during the subsequent pregnancy	Non-GDM during the subsequent pregnancy	p-value
	n = 156	n = 1,484	
**Index pregnancy**			
Tertiary center	87 (55.8)	729 (49.1)	0.114
Maternal age, years old	31.9 ± 4.4	30.5 ± 4.8	<0.001*
Maternal age ≥ 35 years	34 (21.8)	273 (18.4)	0.301
Pre-pregnancy BMI, kg/m^2^	23.0 ± 5.1	20.7 ± 3.1	<0.001*
Pre-pregnancy BMI ≥ 25.0 kg/m^2^	37 (23.7)	102 (6.9)	<0.001*
Smokers	2 (1.3)	15 (1.0)	0.354
Hypertension	4 (2.6)	17 (1.1)	0.137
Hyperthyroidism	1 (0.6)	13 (0.9)	1.000
Hypothyroidism	6 (3.8)	25 (1.7)	0.124
Primiparity	118 (75.6)	1,184 (79.8)	0.224
ART	21 (13.5)	120 (8.1)	0.022*
Gestational body weight gain, kg	10.7 ± 4.2	11.0 ± 3.8	0.387
HDP	35 (22.4)	164 (11.1)	<0.001*
GDM	48 (30.8)	22 (1.5)	<0.001*
Insulin	19/48 (39.6)	2/22 (9.1)	0.010*
Stillbirth ≥ 22 weeks	1 (0.6)	7 (0.5)	0.568
GA at delivery, weeks	39.3 ± 2.0	39.1 ± 2.1	0.333
Preterm birth (<37 weeks)	9 (5.8)	122 (8.2)	0.283
Cesarean section	37 (23.7)	367 (24.7)	0.780
Neonatal sex, male	85 (54.5)	806 (54.3)	0.967
Neonatal height, cm	49.7 ± 3.4	49.3 ± 3.0	0.101
Birthweight, g	3,059 ± 509	2,956 ± 506	0.016*
Heavy for date infant	30 (19.2)	146 (9.8)	<0.001*
Light for date infant	10 (6.4)	141 (9.5)	0.204
Macrosomia (Birthweight ≥ 4 kg)	5 (3.2)	14 (0.9)	0.012*
Placental weight, g	593.0 ± 111.7	567.5 ± 114.6	0.008*
**Pregnancy interval**			
Pregnancy interval, years, median [p25, p75]	2.1 [1.8, 2.7]	2.1 [1.7, 2.5]	0.497
ΔBMI, kg/m^2^	0.86 ± 1.73	0.40 ± 1.35	0.001*
Anuual BMI change, kg/m^2^/year	0.44 ± 1.04	0.19 ± 0.76	0.004*
Annual BMI change			
Weight loss (< 0 kg/m^2^/year)	42 (26.9)	441 (29.7)	┐
0 to < 0.2 kg/m^2^/year	21 (13.5)	388 (26.1)	│
0.2 to < 0.6 kg/m^2^/year	41 (26.3)	375 (25.3)	<0.001*
0.6 to < 1.0 kg/m^2^/year	24 (15.4)	153 (10.3)	│
≥1.0 kg/m^2^/year	28 (17.9)	127 (8.6)	┘
**Subsequent pregnancy**			
Maternal age, years old	34.3 ± 3.6	32.7 ± 5.0	<0.001*
Pre-pregnancy BMI, kg/m^2^	23.8 ± 5.0	21.1 ± 3.3	<0.001*
Pre-pregnancy BMI ≥ 25.0 kg/m^2^	50 (32.1)	138 (9.3)	<0.001*
High parity (Parity ≥ 2)	38 (24.4)	327 (22.0)	0.507
ART	19 (12.2)	120 (8.1)	0.093
Gestational body weight gain, kg	8.5 ± 3.9	10.3 ± 3.6	<0.001*
HDP	23 (14.7)	112 (7.5)	0.002*
GDM	156 (100)	0 (0.0)	–
Insulin	30/156 (19.2)	–	–
Stillbirth ≥ 22 weeks	0 (0.0)	1 (0.1)	1.000
GA at delivery, weeks	39.0 ± 1.5	39.0 ± 1.6	0.879
Preterm birth (<37 weeks)	9 (5.8)	77 (5.2)	0.757
Cesarean section	44 (28.2)	354 (23.9)	0.228
Neonatal sex, male	86 (55.1)	756 (50.9)	0.398
Neonatal height, cm	49.9 ± 2.2	49.7 ± 2.3	0.167
Birthweight, g	3,135 ± 482	3,032 ± 429	0.011*
Heavy for date infant	29 (18.6)	154 (10.4)	0.004*
Light for date infant	5 (3.2)	56 (3.8)	0.646
Macrosomia (Birthweight ≥ 4 kg)	3 (1.9)	16 (1.1)	0.416
Placental weight, g	607.7 ± 113.8	578.4 ± 109.7	0.002*

GDM, gestational diabetes mellitus; BMI, body mass index; DM, diabetes mellitus; ART, assisted reproductive technology; GA, gestational age; HDP, hypertensive disorders of pregnancy.

Data are presented as means ± standard deviation or median [p25, p75] for continuous variables and n (%) for discrete variables. *Statistically significant.

Data are presented as means ± standard deviations or medians [p25, p75] for continuous variables and numbers (percentages) for categorical variables. Statistical significance was set at a *p*-value of <0.05. The statistical analyses were conducted using SPSS version 28.0 for Windows software (SPSS, Inc., Chicago, IL, USA).

## Results

### Participants

A total of 2,219 pregnant women (tertiary centers, n=1,252; primary maternity care units, n=967) were included. Among them, 579 were excluded because of multiple pregnancies (n=379), overt DM (n=27), stillbirth before 22 weeks of gestation (n=66), and missing data on the pre-pregnancy BMI (n=8) and GDM status (n=99) during the index and subsequent pregnancy (**Figure 1**). The remaining 1,640 pregnant women (tertiary centers, n=816; primary maternity care units, n=824) were finally included.

Four women who developed GDM during the index pregnancy developed postpartum DM, and their subsequent pregnancies were treated as overt DM. They were excluded from the study population; their clinical data are listed in **Supplementary Table 1**. Two women needed insulin use during their index pregnancies (cases 2 and 3). One patient did not need insulin for GDM, and her pre-pregnancy BMI was within the normal range (case 1).

### Comparison of Clinical Parameters Between the GDM and Non-GDM Groups

GDM occurred in 70/1,640 women (4.3%) during the index pregnancy and 156/1,640 women (9.5%) during the subsequent pregnancy; 55.8% of the patients with GDM during the subsequent pregnancy were treated at tertiary centers (**Table 1**).

Regarding the index pregnancy characteristics, the following factors were significantly different between the GDM during the subsequent pregnancy and non-GDM during the subsequent pregnancy groups: maternal age (31.9 ± 4.4 *vs*. 30.5 ± 4.8 years, respectively; *p*<0.001), pre-pregnancy BMI (23.0 ± 5.1 *vs*. 20.7 ± 3.1 kg/m^2^, respectively; *p*<0.001), placental weight (593.0 ± 111.7 *vs*. 567.5 ± 114.6 g, respectively; *p*=0.008), incidence of ART conception (13.5% *vs*. 8.1%, respectively; *p*=0.022), hypertensive disorders of pregnancy (HDP) (22.4% *vs*. 11.1%, respectively; *p*<0.001), GDM (30.8% *vs*. 1.5%, respectively; *p*<0.001), having a heavy-for-date infant (19.2% *vs*. 9.8%, respectively; *p*<0.001), and macrosomia (3.2% *vs*. 0.9%, respectively; *p*=0.012). Additionally, the proportion of patients with GDM who used insulin during the index pregnancy was also higher in the GDM during the subsequent pregnancy group than in the non-GDM group (39.6% *vs*. 9.1%, respectively; *p*=0.010).

The median pregnancy interval did not differ significantly between the GDM and non-GDM groups (both 2.1 years, *p*=0.497). In contrast, the ΔBMI and annual BMI change were significantly higher in the GDM group than in the non-GDM group (0.86 ± 1.73 *vs*. 0.40 ± 1.35 kg/m^2^, *p*=0.001; and 0.44 ± 1.04 *vs*. 0.19 ± 0.76 kg/m^2^/year, *p*=0.004, respectively).

### Risk Factors for GDM During the Subsequent Pregnancy

According to the multivariable analysis (**Table 2**), three variables (pre-pregnancy BMI of ≥25.0 kg/m^2^, GDM during the index pregnancy, and an annual BMI change during the pregnancy interval) were significantly associated with GDM during the subsequent pregnancy after adjusting for known risk factors. GDM during the index pregnancy showed the highest aOR for GDM during the subsequent pregnancy [aOR, 26.22; 95% confidence interval (CI), 14.93–46.07]. Therefore, further analysis was performed by stratifying by the presence or absence of GDM during the index pregnancy.

**Table 2:** Univariable and multivariable logistic regression analysis of variables potentially associated with GDM during the subsequent pregnancy.

	n/N (%)	Crude OR	95%CI	p-value	Adjusted OR	95%CI	p-value
Maternal age ≥ 35 years^☨^	34/307 (11.1)	1.24	(0.83-1.85)	0.301	1.12	(0.70-1.78)	0.643
Pre-pregnancy BMI ≥ 25.0^☨^	37/139 (26.6)	4.21	(2.76-6.41)	<0.001*	2.65	(1.61-4.36)	<0.001*
GDM^☨^	48/70 (68.6)	29.54	(17.19-50.74)	<0.001*	26.22	(14.93-46.07)	<0.001*
Macrosomia^☨^	5/19 (26.3)	3.48	(1.24-9.79)	0.018*	2.08	(0.60-7.22)	0.249
Pregnancy interval, years	–	1.13	(0.96-1.33)	0.131	1.10	(0.92-1.32)	0.276
Anuual BMI change, kg/m^2^/year	–	1.42	(1.18-1.71)	<0.001*	1.48	(1.22-1.81)	<0.001*
High parity (Parity ≥ 2)	38/365 (10.4)	1.14	(0.78-1.68)	0.507	1.18	(0.77-1.81)	0.459

n/N: The number of GDM events during the subsequent pregnancy/the number of patients for each variables; OR, odds ratio; CI, confidence interval; BMI, body mass index; GDM, gestational diabetes mellitus.

^☨^Variables during the index pregnancy. *Statistically significant.

The aOR for GDM recurrence during the subsequent pregnancy was calculated in patients who had GDM during the index pregnancy (n=70) (**Table 3**, subgroup 1). In this subgroup, the recurrence rate of GDM was 68.6% (48/70). The annual BMI change and pregnancy interval were not significantly associated with GDM recurrence (aOR, 1.16; 95% CI, 0.75–1.79; and aOR, 1.10; 95% CI, 0.60–2.01; respectively); however, a pre-pregnancy BMI of ≥25.0 kg/m^2^ and insulin use during the index pregnancy were significant (aOR, 5.83; 95% CI, 1.33–25.52; and aOR, 6.98; 95% CI, 1.38–35.38; respectively).

**Table 3:** Subgroup analysis: Univariable and multivariable logistic regression analysis of factors potentially associated with GDM during the subsequent pregnancy.

	Subgroup1: GDM during the index pregnancy								Subgroup2: Non-GDM during the index pregnancy							
	n = 70								n = 1,570							
	N	n/N	cOR	95%CI	p-value	aOR	95%CI	p-value	N	n/N	cOR	95%CI	p-value	aOR	95%CI	p-value
	(%)	(%)							(%)	(%)						
Maternal age ≥ 35 years^☨^	17	14/17	2.61	(0.66-10.24)	0.170	1.86	(0.41-8.41)	0.423	290	20/290	1.00	(0.61-1.66)	0.990	1.00	(0.59-1.67)	0.990
	(24.3)	(82.4)							(18.5)	(6.9)						
Pre-pregnancy BMI ≥ 25.0^☨^	23	20/23	4.52	(1.18-17.38)	0.028*	5.83	(1.33-25.52)	0.019*	116	17/116	2.57	(1.47-4.49)	<0.001*	2.28	(1.28-4.04)	0.005*
	(32.9)	(87.0)							(7.4)	(14.7)						
Insulin use^☨^	21	19/21	6.55	(1.37-31.32)	0.019*	6.98	(1.38-35.38)	0.019*	0	–	–	–	–	–	–	–
	(30.0)	(90.5)							(0.0)							
Macrosomia^☨§^	2	2/2	–	–	–	–	–	–	17	3/17	2.96	(0.84-10.45)	0.093	1.99	(0.53-7.46)	0.305
	(2.9)	(100)							(1.1)	(17.6)						
Pregnancy interval, years	–	–	1.03	(0.69-1.54)	0.893	1.16	(0.75-1.79)	0.513	–	–	1.07	(0.88-1.31)	0.493	1.13	(0.92-1.37)	0.241
Anuual BMI change, kg/m^2^/year	–	–	1.00	(0.64-1.58)	0.986	1.10	(0.60-2.01)	0.765	–	–	1.60	(1.30-1.98)	<0.001*	1.57	(1.27-1.95)	<0.001*
High parity (Parity ≥ 2)^§^	15	15/15	–	–	–	–	–	–	350	23/350	0.94	(0.58-1.51)	0.797	0.93	(0.57-1.50)	0.755
	(21.4)	(100)							(22.3)	(6.6)						

N: The number of patients for each variables; n/N: The number of GDM events during the subsequent pregnancy/N; cOR, clude odds ratio; aOR, adjusted odds ratio; CI, confidence interval; BMI, body mass index; GDM, gestational diabetes mellitus.

^☨^Parameteres of the index pregnancy. ^§^ORs were not calculated because there were no non-GDM during the subsequent pregnancy in subgroup1. *Statistically significant.

**Figure 3:**
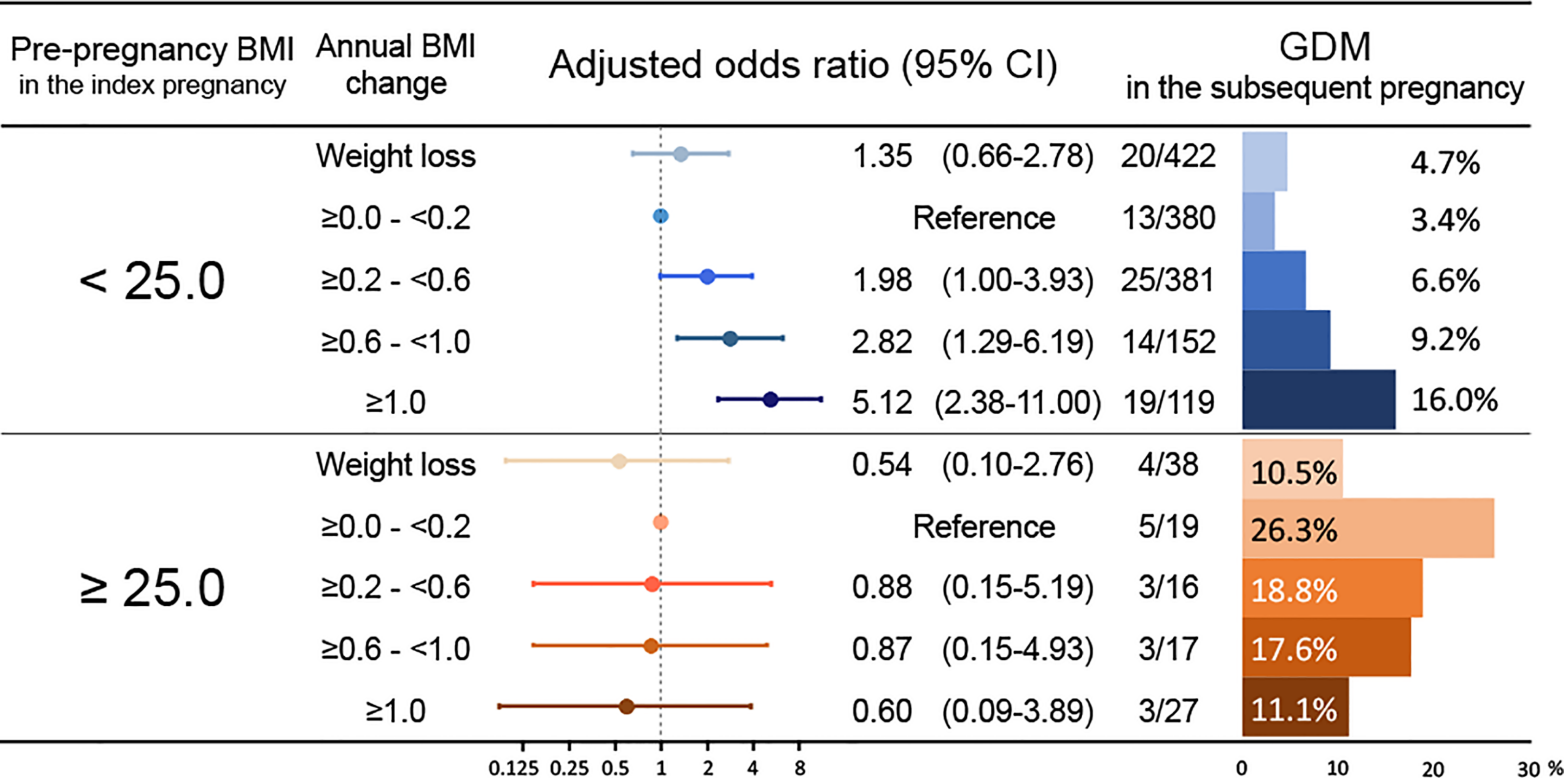
Adjusted odds ratios for GDM during the subsequent pregnancy among women without a history of GDM according to the annual BMI change and pre-pregnancy BMI during the index pregnancy. The multivariable models were adjusted for maternal age of ≥35 years, pre-pregnancy BMI in the index pregnancy, pregnancy interval, and classified annual BMI changes. The forest plot represents the adjusted odds ratio for the classified annual BMI changes for GDM during the subsequent pregnancies. The bar chart displayed on the right shows the incidence of GDM during the subsequent pregnancy according to the degree of annual BMI change. The number of GDM events during the subsequent pregnancy/the total number is shown on the left of the bar chart. BMI, body mass index; GDM, gestational diabetes mellitus; CI, confidence interval.

In the subgroup of patients without a history of GDM during the index pregnancy (n=1,570) (**Table 3**, subgroup 2), 108 women (6.9%) developed GDM during the subsequent pregnancy. The annual BMI change was associated with GDM during the subsequent pregnancy (aOR, 1.57; 95% CI, 1.27–1.95; **Table 3**), and a pre-pregnancy BMI of ≥25.0 kg/m^2^ during the index pregnancy was also associated with GDM during the subsequent pregnancy (aOR, 2.28; 95% CI, 1.28–4.04; **Table 3**). In this subgroup, the aORs for GDM during the subsequent pregnancy were calculated using the five categories of annual BMI changes, with the reference category being 0.0–0.2 kg/m^2^/year (**Figure 3**). Among women with a pre-pregnancy BMI of <25.0 kg/m^2^ during the index pregnancy, those with BMI gains of ≥0.6–<1.0 units/year and ≥1.0 kg/m^2^/year had a 2.82 (95% CI, 1.29–6.19) and 5.12 (95% CI, 2.38–11.00) higher odds of GDM during the subsequent pregnancy, respectively. On the other hand, among women with a pre-pregnancy BMI of ≥25.0 kg/m^2^ during the index pregnancy, none of the five categories of annual BMI change were significantly associated with GDM during the subsequent pregnancy. Although no significant difference was detected, the weight loss category showed a trend to reduce the prevalence of GDM compared to the reference category (10.5% *vs*. 26.3%, **Figure 3**). Additionally, increasing annual BMI gain showed an inverse trend with GDM prevalence and aOR. In the further multivariable analysis of this subgroup (**Supplementary Table 2**), annual BMI was not an independent factor, but pregnancy interval was independently associated with GDM during the subsequent pregnancy (aOR 1.72, 95% CI 1.12–2.63). In this subpopulation, increasing annual BMI gain also showed a shorter trend of pregnancy interval (**Supplementary Figure 1**), similar to a trend of GDM prevalence (shown in **Figure 3**).

## Discussion

This was the first study to evaluate the association between GDM during the subsequent pregnancy with the annual BMI change during the interpregnancy period. Annual BMI gain during the interpregnancy period was an independently associated with GDM during subsequent pregnancies. Higher pre-pregnancy BMI, and GDM during the index pregnancy were also factors which were independently associated with GDM during the subsequent pregnancy. Among these factors, a history of GDM during the index pregnancy was the most significantly associated with the GDM during the subsequent pregnancy. The 68.6% (48/70) of women with a history of GDM experienced recurrent GDM during the subsequent pregnancy, and the recurrence rate was as high as almost 90% in patients with GDM who had a pre-pregnancy BMI of ≥25.0 kg/m^2^ during the index pregnancy. However, the annual BMI change during the interpregnancy period was not significantly associated with recurrent GDM. On the other hand, in women without a history of GDM, the annual BMI gain was associated with GDM during the subsequent pregnancy. Furthermore, an annual BMI gain of ≥0.6 kg/m^2^/year during the interpregnancy period was associated with GDM during the subsequent pregnancy among women with a pre-pregnancy of BMI of <25.0 kg/m^2^ and without development of GDM during the index pregnancy.

Previous studies have suggested that a history of GDM and insulin use were risk factors for GDM during the subsequent pregnancy (3, 4, 25, 27). The recurrence rate in this study was consistent with those of previous studies (3, 5, 53). It is important to note that patients with a history of GDM are at a high risk of developing GDM during the subsequent pregnancy. While parity is also correlated with the risk of GDM (51), approximately 70% of the patients with GDM during the subsequent pregnancy did not have GDM during the index pregnancy, suggesting that focusing only on those who had GDM during the index pregnancy would not reduce the incidence of GDM during the subsequent pregnancy. Other known risk factors for GDM development during the subsequent pregnancy that have been reported are as follows: older maternal age, higher pre-pregnancy BMI, and higher interpregnancy weight gain (4, 27, 28); these were consistent with the findings of the present study.

Using subgroup analyses, the present study identified the subgroup at risk of interpregnancy BMI gains, which in turn could increase the risk of GDM in subsequent pregnancies. In women with a pre-pregnancy BMI of <25.0 kg/m^2^ and without a history of GDM during the index pregnancy, interpregnancy BMI gains were significantly correlated with the incidence of GDM during the subsequent pregnancy. The cut-off value of the annual BMI change was found to be ≥0.6 kg/m^2^/year according to the multivariable analysis. In this study population, the mean annual BMI change was approximately 0.21 kg/m^2^/year, which was comparable to those reported in previous studies (34, 35). Thus, maintaining an annual weight gain of <0.6 kg/m^2^/year might be advisable for women with a pre-pregnancy BMI of <25.0 kg/m^2^ and without a history of GDM during the index pregnancy to prevent GDM occurrence during the subsequent pregnancy; however, most clinicians have not paid much attention to those women. Additionally, weight loss during interpregnancy might not reduce GDM risk during the subsequent pregnancy for those women (**Figure 3**).

On the other hand, the annual BMI change was not significantly associated with GDM during the subsequent pregnancy in the following subgroups: women whose pre-pregnancy BMI was ≥25.0 kg/m^2^ and didn’t have GDM during the index pregnancy, and those who had GDM during the index pregnancy. For the former subgroup, we speculated that they might be resistant to GDM development due to BMI gain. Some specific variants might be related to this resistance, as several genetic variants have decreased GDM risk (54). The multivariable analysis in this subgroup showed pregnancy interval as an independent risk for GDM during the subsequent pregnancy. It suggested that ‘aging’ might be more critical than ‘BMI gain’. The paradoxical trend, which was not statistically significant, that higher annual BMI gain categories had lower prevalence of GDM during the subsequent pregnancies (**Figure 3**), would depend on higher annual BMI gain categories with shorter pregnancy intervals (**Supplementary Figure 1**). Additionally, weight loss might reduce the risk of GDM in the subsequent pregnancy for these women. No significant difference was detected, but this might have been due to the low number of the weight-loss population in this present study. For the latter subgroup, it is worth noting that in this study, 87% (20/23) of the patients with GDM who had a pre-pregnancy BMI of ≥25.0 kg/m^2^ during the index pregnancy experienced recurrent GDM during the subsequent pregnancy. However, the importance of interpregnancy care for these patients should not be overlooked. Another retrospective study suggested that interpregnancy weight loss might reduce the risk of GDM during the subsequent pregnancy among overweight patients who had GDM during the index pregnancy (28). Some active interventions to lose weight might be more effective for these patients, and further prospective research is needed. More evidence for interpregnancy care protocols to prevent GDM is warranted. The present study was the first to demonstrate the association between annual BMI gain during the interpregnancy period and GDM incidence during subsequent pregnancies among women with or without GDM during the index pregnancy.

## Strengths and Limitations

This study had several strengths. First, this was the first study to assess the association between GDM during the subsequent pregnancy and annual BMI changes during the pregnancy interval. Second, the aOR for GDM was also stratified by several other factors, including a history of GDM and pre-pregnancy BMI during the index pregnancy. Third, as this was a multicenter study, both primary maternity care units and tertiary care centers participated in this study. The study population included pregnant women at various risk levels, which helped minimize selection bias. Recent studies on the risk of recurrent GDM have included only women who gave birth at tertiary centers (4, 26, 27). The data used in this study were detailed and reliable, as required by the national registry studies.

This study also had several limitations. First, the study population consisted only of patients who had both index and subsequent pregnancy records available. The following patients were excluded: women who delivered a subsequent baby at a non-participating institute, those who had an abortion in a subsequent pregnancy, and those who developed infertility after the index pregnancy. These populations might have other problems; however, these were outside the scope of our study. Second, we did not follow up on the postpartum weights. The annual BMI change was not measured as a part of an annual check but was calculated according to the pregnancy interval and ΔBMI. However, the mean weight change from pre-pregnancy to 1 year after delivery, which is approximately 2 years, has been reported to be 0.9 kg (55), which was comparable to the age-related weight gain (33–35). Therefore, the difference between actual annual BMI change and calculated annual BMI change would be not so significant because the mean pregnancy interval was 2.1 years. Third, only 61.3% of the patients (1,006/1,640) were verified their family history of diabetes, and most of the patients who had an unknown family history of diabetes were patients in tertiary centers (548/634 [86.4%]). Therefore, we thought its inclusion in the analysis would make a reliable assessment difficult even though it was a possible confounder (56). Additionally, the women who had systemic diseases interfering with glucose homeostasis were not excluded from the analysis in this study. The risk of developing GDM during the subsequent pregnancies was analyzed separately by stratifying according to the presence or absence of GDM during the index pregnancy, regardless of underlying disease or genetic background. We have speculated that some women with such complications might have developed GDM during the index pregnancy and treated as women with a history of GDM during the index pregnancy. These have limitations in terms of accurate risk assessment, but when considering future applications in interpregnancy care, it will be an advantage in terms of simplifying the assessment of the patients. Fourth, self-reported weight was used to calculate BMI. However, most participants measured their weights at the prenatal visit in the first trimester, so the difference between the self-reported and actual weight is likely to be minimal.

Interpregnancy health checks, including weight checks for women who hope to have subsequent pregnancies, have not been provided in clinical settings in Japan. Based on the current results, the maintenance of an appropriate annual BMI change may be advised. However, it is still unclear whether active interventions can prevent GDM in subsequent pregnancies. Thus, we plan to implement such interventions based on this study’s findings. Finally, the subgroup with a history of GDM was a small population, and research with more extensive populations is warranted to confirm the results.

In conclusion, in this study, an annual weight gain of ≥0.6 kg/m^2^/year was independently associated with higher incidence of GDM during the subsequent pregnancy in patients with a pre-pregnancy BMI of <25.0 kg/m^2^ and without a history of GDM during the index pregnancy. Furthermore, patients with a history of GDM and insulin use during the index pregnancy had higher incidence of GDM during the subsequent pregnancy. However, the association between annual BMI change and GDM incidence during the subsequent pregnancy was not confirmed in this subgroup.

These results might help lay the foundation for further research to determine whether limiting annual BMI gains can prevent GDM during a subsequent pregnancy and establish protocols for interpregnancy care to prevent GDM. Preventing GDM will in turn help improve the health outcomes of women and their children.

## Data Availability Statement

The raw data supporting the conclusions of this article will be made available by the authors, upon reasonable request, and with the permission of Kishokai Medical Corporation.

## Ethics Statement

The studies involving human participants were reviewed and approved by the ethics committee of Nagoya University Hospital (approval number: 2015–0415) in accordance with the Declaration of Helsinki. The ethics committee waived the requirement for written informed consent because of the retrospective nature of the study.

## Author Contributions

ST, TK, and MYo conceived the study. ST, TK, and FK conducted the statistical analyses. ST, TK, TU, KI, TN-K, YM, YI, SY, MYa, YK, HO, and HK collected and interpreted the clinical data. ST and TK drafted the manuscript. All authors contributed to the interpretation of the results and approved the final manuscript.

## Conflict of Interest

Authors SY and MYa are an employee, and President and CEO of Kishokai Medical Corporation, respectively.

The remaining authors declare that the research was conducted in the absence of any commercial or financial relationships that could be construed as a potential conflict of interest.

## Publisher’s Note

All claims expressed in this article are solely those of the authors and do not necessarily represent those of their affiliated organizations, or those of the publisher, the editors and the reviewers. Any product that may be evaluated in this article, or claim that may be made by its manufacturer, is not guaranteed or endorsed by the publisher.

## Supplementary Material

Supplementary Table 1 Detailed characteristics of individuals diagnosed with DM after the index pregnancy

Supplementary Table 2 Univariable and multivariable logistic regression analysis of variables potentially associated with GDM during the subsequent pregnancy among women whose pre-pregnant BMI were ≥25.0 kg/m^2^ and didn’t have GDM during the index pregnancy.

Supplementary Figure 1 Mean pregnancy interval for each annual BMI change categories.
