# Supplementary figures and images for: Annual Body Mass Index Gain and Risk of Gestational Diabetes Mellitus in a Subsequent Pregnancy
Source: Front Endocrinol (Lausanne). 2022 Mar 25;13:815390. doi: 10.3389/fendo.2022.815390 (PMC8990746; doi:10.3389/fendo.2022.815390)

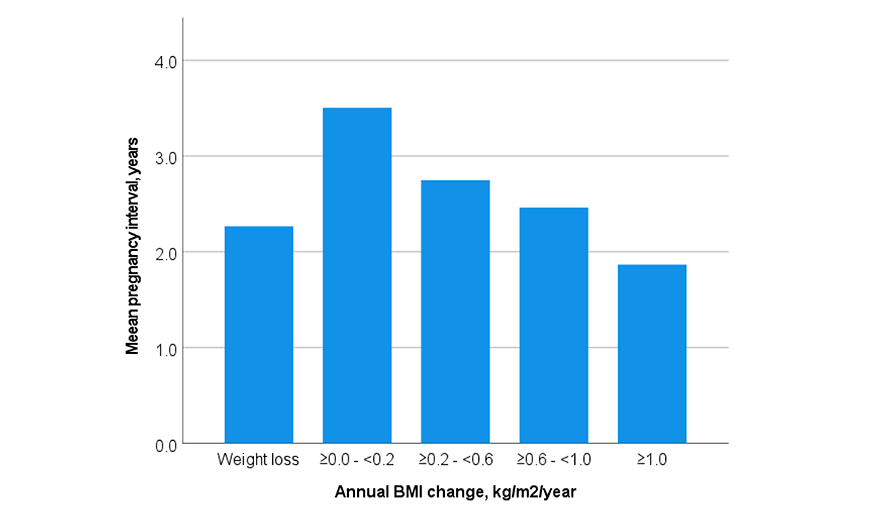

Supplement: Supplementary Figure 1 — Mean pregnancy interval for each annual BMI change categories. [file Image_1.tif]
